# Culturable Seed Microbiota of *Populus trichocarpa*

**DOI:** 10.3390/pathogens10060653

**Published:** 2021-05-24

**Authors:** Sabrina Heitmann, Gillian E. Bergmann, Edward Barge, Mary Ridout, George Newcombe, Posy E. Busby

**Affiliations:** 1 Department of Botany and Plant Pathology, Oregon State University, Corvallis, OR 97331, USA; heitmans@oregonstate.edu (S.H.); gbergmann@ucdavis.edu (G.E.B.); bargee@oregonstate.edu (E.B.); 2 Department of Forest, Rangeland and Fire Sciences, University of Idaho, Moscow, ID 83844-1133, USA; mridout@uidaho.edu (M.R.); georgen@uidaho.edu (G.N.)

**Keywords:** seed microbes, *Pseudomonas syringae*, *Cladosporium*, *Aureobasidium*, *Diaporthe*, *Alternaria*

## Abstract

Plants harbor a diverse community of microbes, whose interactions with their host and each other can influence plant health and fitness. While microbiota in plant vegetative tissues has been extensively studied, less is known about members of the seed microbiota. We used culture-based surveys to identify bacteria and fungi found in the seeds of the model tree, *Populus trichocarpa*, collected from different sites. We found that individual *P. trichocarpa* seeds typically contained zero or one microbe, with common taxa including species of *Cladosporium*, *Aureobasidium*, *Diaporthe*, *Alternaria*, and *Pseudomonas,* a bacterium. *Pseudomonas* isolates were associated with seed mortality and were negatively associated with the occurrence of fungal isolates within *Epicoccum*, *Alternaria*, and *Aureobasidium* from the same seed. Next, we conducted an inoculation experiment with one of the isolated seed microbes, *Pseudomonas syringae pv. syringae*, and found that it reduced seed germination and increased seedling mortality for *P. trichocarpa*. Our findings highlight common fungi and bacteria in the seeds of *P. trichocarpa*, prompting further study of their functional consequences. Moreover, our study confirms that *P. syringae pv. syringae* is a seed pathogen of *P. trichocarpa* and is the first report that *P. syringae pv. syringae* is a lethal seedling pathogen of *P. trichocarpa*, allowing for future work on the pathogenicity of this bacterium in seedlings and potential antagonism with other seed microbes.

## 1. Introduction

Plants are colonized by a diverse array of microorganisms that influence host health and productivity [1]. While some microbes, like pathogens, can be detrimental to host plants, many microbes can benefit plants by increasing disease resistance [2], nutrient uptake [3] or growth [4]. The effects of both pathogenic and beneficial microbes are particularly important to plants at the seed to seedling stages, when rates of mortality are high [5]. However, the vast majority of research on plant microbiota has focused on vegetative plant tissues, leaving us with an incomplete understanding of the taxonomic and functional diversity of seed microbiota.

As the reproductive unit of a plant, seeds represent a vital stage in a plant’s life cycle. Seeds are the source of genetic diversity and units of dispersal that influence plant community interactions and distribution [6]. However, seedling establishment is a natural bottleneck, with mortality caused by herbivory, drought, and pathogens [7]. Some seed microbes can improve the health and fitness of a seedling during this challenging life stage. Microbial seed research has primarily focused on vertically-transmitted clavicipitaceous seed endophytes which have been shown to prevent herbivory [8], reduce the effects of drought stress [9], and increase host biomass [10].

More recently, interest in seed microbes of cultivated crops has heightened for applications in sustainable crop production. Seedborne microbes can be applied to aid seedling development [11], increase resistance against disease [12], and mitigate stress across the host generation [13]. Due to the impact that seed microbiota can have on plant health, it is important to understand the factors influencing the seed microbial community composition in order to integrate seed microbiota in agricultural settings. Previous studies have found that deterministic community assembly plays a large role in shaping seed microbial communities. Local site conditions [14], plant genotype or cultivar [15,16], and management practices, such as seed processing and storage [17], can all influence the assembly of seed microbiota.

Despite their possible impact on forest and plantation health, less is known about the factors that shape the seed microbiota of trees. In a study that analyzed the drivers of seed microbiota in nine tree species across Europe and North America, the host species explained roughly twice as much (33–35%) of the fungal community composition compared to the site (17%) [18]. In *Quercus petraea*, fungal communities in seeds were primarily shaped by maternal effects and to a lesser extent, the biotic microenvironmental conditions (i.e., leaf litter and soil) [19]. Both pathogenic and microparasitic seed fungi were identified, which suggests that seeds can be the vectors of both pathogenic and beneficial microbiota.

The black cottonwood (*Populus trichocarpa* Torr. & Gray) is a model tree for studying plant-associated microbiota [20] with leaf, stem, root and soil-associated microbiota already well characterized [21,22,23]. However, no study has holistically examined the microbes of the *P. trichocarpa* seeds produced by female trees in this dioecious species. One explanation for this may be that *P. trichocarpa* is almost exclusively propagated by branch cuttings, and thus seed pathogens are not an agricultural concern. However, seedlings are essential to natural riparian regeneration, and new research has identified a seed pathogen of *P. trichocarpa*, *Pseudomonas syringae pathovar. syringae* [24], prompting interest in understanding its frequency and distribution in wild tree populations.

*Pseudomonas syringae* is a ubiquitous bacterium that has primarily been studied as a plant pathogen. *P. syringae* pathovars are typically host-specific and cause a range of diseases in almost all economically important agricultural crops [25]. Infections of *Populus spp.* by *P. syringae* have previously been reported, and can cause necrosis due to the pathogen’s ice nucleating abilities [26,27]. The newly identified *P. syringae pv. syringae* seed pathogen has been shown to cause lesions on *P. trichocarpa* leaves and decrease *P. trichocarpa* germination rates [24], but its pathogenicity on seedlings has not been evaluated. Moreover, the distribution and co-occurrence patterns of *P. syringae pv. syringae* with other seed microbes is unclear.

The objective of our study was to characterize culturable seed microbes—fungi and bacteria—from individual seeds of *P. trichocarpa* trees collected from different sites. We paid particular attention to the abundance and distribution of the recently identified seed pathogen, *P. syringae pv. syringae*. In total, we identified culturable microbes from the seeds of 32 *P. trichocarpa* trees growing in three different environments (Westport, Oregon, USA; Moscow, Idaho, USA; and Clearwater River, Idaho, USA) and from two different study years (2017, 2018). We then conducted an inoculation experiment to test the pathogenicity of one of our cultured *P. syringae pv. syringae* isolates (NP10-3) [24].

## 2. Results

### 2.1. Description of Populus trichocarpa Seed Microbiota

**Figure 1:**
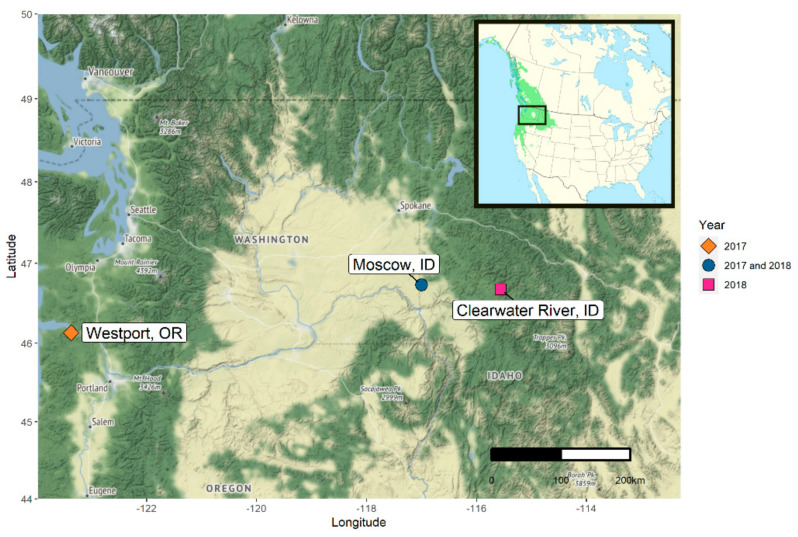
The locations and years of seed sampling efforts are shown: Westport, Oregon (yellow), Moscow, Idaho (blue), and Clearwater River, Idaho (pink) and inset map of Canada and the United States with the geographic range of Populus trichocarpa in green.

To characterize the *P. trichocarpa* seed microbiota, seed microbes were collected from individual trees in several different sites and in two different years (Figure 1). In 2017, we sampled eight trees from a common garden in Westport, Oregon (Oregon 2017), and a single tree in Moscow, Idaho (Idaho 2017). In 2018, we sampled the same tree from Moscow, Idaho in addition to 21 trees from along the Clearwater River in Idaho (Idaho 2018). The Idaho sites are c. 48 to 60 km apart; open-pollinated seeds from each sampled tree comprised a half-sibship. Due to the variation in our opportunistic sampling efforts and isolation methods across study sites and years, we focus on reporting patterns observed within each of the datasets rather than making direct comparisons between or among the datasets.

#### 2.1.1. Oregon 2017

**Figure 2:**
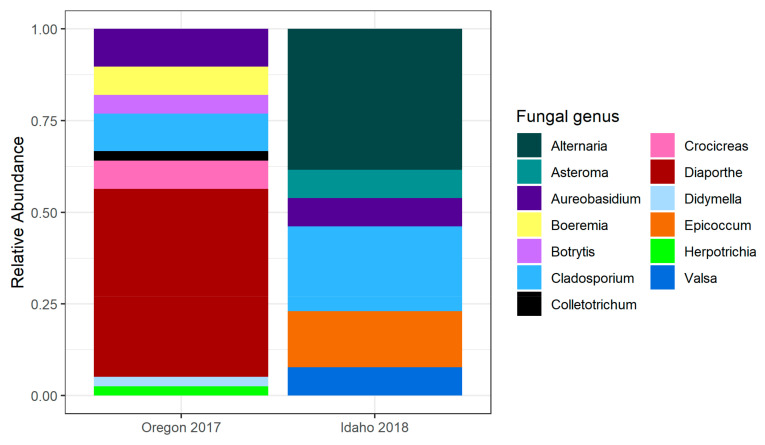
Relative abundance of all sequenced fungi from Westport, Oregon (trees = 8, isolates = 39), Oregon 2017 sampling effort and sequenced fungal morphotypes from Clearwater River, Idaho (trees = 12, isolates = 12), and Moscow, Idaho (tree = 1, isolate = 1), Idaho 2018 sampling effort. The Moscow, Idaho, isolate was identified as *Alternaria*.

In the Oregon 2017 trees, either zero or one culturable microbe emerged from individual seeds. While we found no bacteria, fungi were isolated from 46 of the 800 seeds (5.8%) (Table 1). The 39 successfully sequenced fungal isolates (Table S1) belonged to three classes within *Ascomycota*: *Sordariomycetes* (53.8%), *Dothideomycetes* (33.3%), and *Leotiomycetes* (12.8%). More specifically, the species within *Diaporthe* (51.3%), *Cladosporium* (10.3%), and *Aureobasidium* (10.3%) were most abundant (Figure 2).

#### 2.1.2. Idaho 2017

Most of the seeds collected from the single sampled tree contained zero microbes (84%); the remainder contained one (15.3%) or two (0.8%). Fungi emerged from 56 out of 1050 seeds (5.3%), with isolates morphologically identified as *Cladosporium* (46.8%), *Aureobasidium* (13.6%) and *Alternaria* (5.1%). Bacteria emerged from 120 of 1050 seeds (11.4%), and fungi and bacteria co-occurred in five total seeds (0.5%). The majority of bacterial isolates (84.3%) were morphologically identified as *Pseudomonas*. The remaining bacterial isolates (15.7%) were not identified. *Pseudomonas* were found to be associated with seed mortality (X^2^ = 379.71, *p* < 0.001).

#### 2.1.3. Idaho 2018

The seeds collected from Clearwater River, ID, in 2018, contained zero microbes (56.7%), one microbe (37.9%), or two microbes (5.3%). Fungi emerged from 260 out of 1260 seeds (20.6%), and bacteria emerged from 353 out of 1260 seeds (28.0%), with fungi and bacteria co-occurring in 51 of those seeds (4.1%). *Cladosporium* and *Alternaria* exhibited the greatest relative abundance of the morphologically identified fungi (36.7% and 35.2%, respectively).

Most seeds collected from the single tree in Moscow, ID, in 2018, contained one microbe (63.3%); the remaining contained zero (18.3%) or two or more (18.3%). Fungi emerged from 15 out of 60 seeds (25%) and bacteria emerged from 46 out of 60 seeds (76.7%), with fungi and bacteria co-occurring in nine of those seeds (15%). Fungi were morphologically identified as *Cladosporium* (86.7%) and *Alternaria* (13.3%).

Thirteen representative fungal morphotypes from Idaho, 2018, were sequenced and successfully identified to the genus level (Table S1). The 12 representative fungal morphotypes from Clearwater River, ID, were identified to *Alternaria*, *Asteroma*, *Aureobasidium*, *Cladosporium*, *Epicoccum*, *and Valsa* (Figure 2). One fungal morphotype was sequenced from Moscow, ID and was identified to the genus, *Alternaria*.

**Figure 3:**
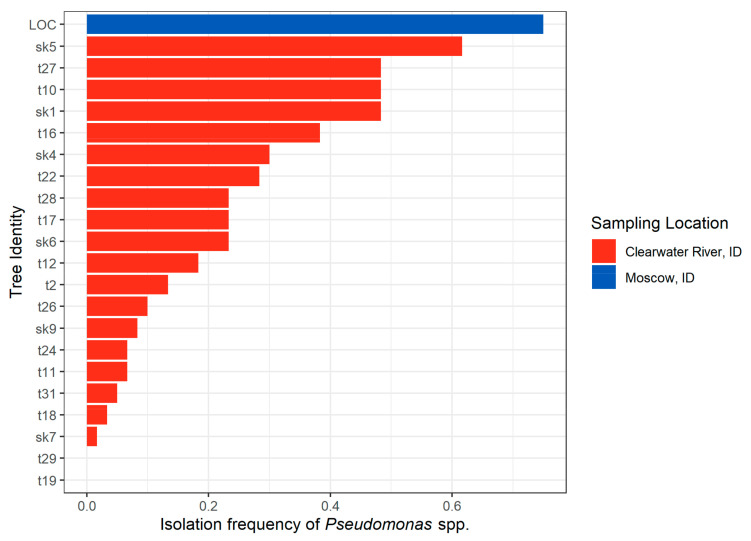
Isolation frequency of *Pseudomonas* across sampled Idaho 2018 trees from Clearwater River riparian communities, Idaho (21 trees), and Moscow, Idaho (one tree).

*Pseudomonas* were isolated from 20 out of the 22 trees surveyed in the Idaho 2018 sampling effort, with an isolation frequency from 0 to 48.5% for Clearwater River, Idaho trees and 75% for the single Moscow, ID tree (Figure 3). *Pseudomonas* comprised 78.2% of all morphologically identified bacteria, while the remaining 21.8% of bacterial isolates were not identified. *Pseudomonas* isolates were associated with seed mortality (X^2^ = 307.66, *p* < 0.001) and were negatively associated with the occurrence of fungal isolates within *Epicoccum* (X^2^ = 11.24, *p* < 0.001), *Alternaria* (X^2^ = 5.67, *p* = 0.012), and *Aureobasidium* (*p* = 0.004) genera from the same seed.

### 2.2. Seed and Seedling Mortality Experiment

Our inoculation experiment confirmed the pathogenicity of *P. syringae pv. syringae* as a seed pathogen and as a lethal seedling pathogen of *P. trichocarpa*. While 50 out of 50 uninoculated seeds germinated, only 35 out of 49 (71%) seeds inoculated with *P. syringae pv. syringae* germinated after two weeks. Uninoculated seedlings also had lower mortality, compared to inoculated seeds; one out of 49 (2%) uninoculated seedlings died, compared to 11 out of 49 (22.4%) inoculated seedlings. Compared to the uninoculated seeds, *P. trichocarpa* seeds exposed to *P. syringae pv. syringae* had a significantly decreased seed germination rate (X^2^ = 16.64, *p* < 0.001) and significantly increased seedling mortality (X^2^ = 9.72, *p* = 0.002).

## 3. Discussion

The primary objective of this study was to characterize the culturable seed microbes of *P. trichocarpa*, with particular attention paid to the newly described *P. syringae pv. syringae* seed pathogen [24]. Our sampling methods differed between years and sites, limiting our ability to make cross-site and cross-year comparisons. However, consistent, notable patterns emerged within each dataset. Across all three datasets, the majority of *P. trichocarpa* seeds collected contained either zero or one culturable microbe per seed. This bottleneck has been additionally reported in *Centaurea stoebe* [28] and 27 different common crop species [29]. More recently, this pattern was confirmed in Newcombe et al. [30], which surveyed the culturable seed microbiota of 98 plant species. This finding is the basis for the primary symbiont hypothesis, which states that seeds typically contain a single dominant microbe that is functionally important. While our limited data supports primary symbionts in seeds, future studies that couple a culture-dependent and culture-independent sequencing approach across a broad range of hosts would be better suited to further substantiate this hypothesis [31].

All morphotyped and sequenced fungi found in *P. trichocarpa* seed were ascomycetes, belonging to Sordariomycetes, Leotiomycetes, and Dothideomycetes. These fungi are taxonomically similar to leaf-associated fungi in *P. trichocarpa*, which commonly include species of *Epicoccum*, *Cladosporium*, and *Alternaria* [32]. This finding suggests that, like leaf microbes, many seed microbes are horizontally transmitted from airborne inoculum. In contrast, seed fungi differ markedly from common rhizosphere fungi [22] and wood fungi [23], supporting previous studies that have demonstrated strong tissue specificity within the plant microbiome [33,34,35].

Surprisingly, we did not recover common fungal seed pathogens, such as *Fusarium* and *Rhizoctonia* [36]. However, fungal genera (*Botrytis*, *Valsa*, *Boeremia*) that are known to cause disease in agricultural crops and woody hosts, including *P. trichocarpa*, were isolated from seeds across sites and years. None of these fungal isolates were immediately lethal after emerging from the seeds, suggesting they are not *P. trichocarpa* seed pathogens, as was found for *P. syringae pv. syringae*.

Bacteria were isolated in higher abundance than fungi in the Idaho 2017 and 2018 seeds. Yet, no bacteria emerged from the Oregon 2017 seeds. Potential causes for this variation could be differences in surface sterilization methods, host genetic or environmental factors. Over 75% of bacteria isolated from the Idaho 2017 and 2018 seeds were morphologically identified as *Pseudomonas*, a functionally diverse bacterial genus containing over 200 species [37]. We found that *Pseudomonas* were negatively correlated with *Alternaria*, *Epicoccum*, and *Aureobasidium* in the Idaho 2018 seeds, which is consistent with exclusionary interactions that are commonly found in seeds [28]. However, when two microbes did co-occur in the same seed, it was almost always with one bacterial and one fungal isolate. Two fungi or two bacteria rarely emerged from the same seed, which could indicate that competitive interactions are microbe-specific.

We observed an association between *Pseudomonas* and mortality in the Idaho 2018 seeds, which led us to further investigate *P. syringae pv. syringae*, a well-known pathogen of mature *P. trichocarpa* trees that has only recently been discovered as a seed pathogen to *P. trichocarpa* [24]. Our study confirms that *P. syringae pv. syringae* is a seed pathogen of *P. trichocarpa* and is the first report that *P. syringae pv. syringae* is a seedling pathogen of *P. trichocarpa*. This knowledge can inform future studies on the success and predictability of *P. trichocarpa* seedling emergence, as it can be influenced by the presence of this widespread seed microbe.

To our knowledge, this study is the first culture-based survey of seed microbes of the model tree, *P. trichocarpa*. Most seeds possessed either zero or one symbiont per seed, with common taxa including species of *Cladosporium*, *Aureobasidium*, *Diaporthe*, *Alternaria*, and *Pseudomonas*. The composition of seed microbiota varied among our three sites, possibly as a result of differences in microbial species pools (i.e., airborne inoculum), abiotic environmental factors, host genetic variation, or other assembly processes. Future efforts are needed to more fully characterize fungi and bacteria using a culture-free method. In addition, manipulative studies will help to elucidate how early arrival into the plant microbiota via the seed impacts the composition and function of the developing plant microbiota.

## 4. Materials and Methods

### 4.1. Sampling Populus trichocarpa Seeds

We sampled culturable microbes from seeds collected directly from the branches of individual, female *P. trichocarpa* trees in several different sites, in two different years (Figure 1). In 2017, we sampled eight trees in Westport, Oregon (Oregon 2017) and a single tree in Moscow, Idaho (Idaho 2017). In 2018, we sampled the same tree from Moscow in addition to 21 trees from along the Clearwater River, Idaho (Idaho 2018).

Seed capsules were collected from branches of eight different ten year old *P. trichocarpa* genotypes located in a cool, wet plantation in Westport, Oregon in June 2017 [38]. We used extendable pruning poles to sample catkins of upper canopy branches containing seed capsules, thus seed capsules were not exposed to the forest floor environment.

In summer 2017, closed *P. trichocarpa* seed capsules were collected from a single tree of unknown age on the University of Idaho campus (a warm, dry site) in Moscow, ID using pruning poles.

In summer 2018, *P. trichocarpa* seeds were obtained by sampling the same Idaho 2017 tree located in Moscow, ID, in addition to twenty-one trees of unknown age found along the Clearwater River, ID. For more details on the climate and environmental conditions of the intermountain region of the Pacific Northwest see Ridout et al. 2017 [39].

### 4.2. Seed Microbe Isolation

For Oregon 2017, seeds capsules were air-dried at room temperature in brown paper bags; within a few days, capsules opened and seeds were released. One hundred seeds per tree (N = 800 total) were surface sterilized by soaking for one minute in 1% hypochlorite with two drops of TWEEN and washed three times with sterile, distilled water for one minute each [32]. Seeds were then plated onto 4% potato dextrose agar (PDA) and incubated for ten days to allow for germination and isolation of seed-associated microbes. Efficacy of the surface sterilization was confirmed by imprinting sterilized seeds onto PDA then removing and monitoring for growth on the media. Seed microbes were isolated into pure culture as they emerged from the germinating seeds. Germination and microbial incidence were recorded.

For Idaho 2017, seeds were removed aseptically from each capsule in a laminar hood, and 1050 seeds were plated onto 4% PDA. Surface sterilization was not used since seeds were removed from capsules under sterile conditions. Seeds were incubated at room temperature for eleven days to allow for seed germination and isolation of seed-associated microbes. Seed microbes were isolated into pure culture as they emerged from seeds. Germination, microbial incidence, and seedling mortality were recorded.

Idaho 2018 followed the same surface sterilization and isolation of seed microbe protocol as Idaho 2017. In this effort, 60 seeds from multiple capsules per tree were plated from each tree. A total of 1260 seeds were plated from 21 trees located in Clearwater River, ID and a total of 60 seeds were plated from the single tree located in Moscow, ID.

### 4.3. Seed Microbe Identification

All Oregon 2017 isolates were identified by DNA sequencing. DNA was extracted by scraping hyphae from the surface of colonized petri plates and extraction and neutralization buffers from the REDExtract-N-Amp Plant DNA Kit (Sigma-Aldrich, Saint Louis, MO, USA) were used for cell lysing. ITS1-F [40] and LR3 [41] primers were used to amplify the internal transcribed spacer (ITS) and the nuclear large subunit (LSU) region of each fungal isolate [42]. The PCR reactions were 50 μL in volume, including 30 μL of GoTag Green Master Mix (Promega Corporation) and 4 μL of genomic DNA. The PCR was run for 95 °C for 3 min followed by 32 cycles of 95 °C for 30 s, 50 °C for 30 s, and 72 °C for 30 s, and finally 72 °C for 5 min and cooled down to 10 °C. We used gel electrophoresis to visualize PCR products. If bands were present, products were sent to MCLAB (San Francisco, California, United States) for sequencing and PCR clean-up using ExoSAP. SeqTrace [43] and GENEIOUS 11.1.5 (http://www.geneious.com, accessed on 12 July 2017) [44] were then used to trim and pair forward and reverse reads. The final reads were used in BLAST queries to determine taxonomic identity. Identified sequences were submitted to Genbank (accessions MT786254.1–MT786305.1).

Idaho 2017 seed bacteria were morphologically identified to the *Pseudomonas* genus (or other). Seed fungi were morphologically identified to one of three genera: *Cladosporium*, *Alternaria*, *Aureobasidium* (or other).

Idaho 2018 seed bacteria were morphologically identified to the *Pseudomonas* genus (or other). Seed fungi collected were morphologically identified to *Cladosporium*, *Alternaria*, *Aureobasidium*, *Epicoccum* (or other). At least one representative isolate of each genus was sequenced for identification following the Oregon 2017 protocol, for a total of 12 sequenced fungal isolates from Clearwater, ID and one sequenced fungal isolate from Moscow, ID. Identified sequences were submitted to Genbank (accessions MT786254.1–MT786305.1).

### 4.4. Seedling Mortality Experiment

While isolating microbes from *P. trichocarpa* seeds, we observed an association between seedling mortality and *Pseudomonas* emergence, a pattern also noted by Saint-Vincent et al. [24]. Thus, we conducted an inoculation experiment to determine if one of our isolates of *P. syringae pv. syringae* influenced seed germination and seedling mortality. *P. syringae pv. syringae* inoculum was prepared by streaking a fresh culture onto 4% PDA, which was then grown for 3 days until *P. syringae pv. syringae* covered the entire plate. *P. trichocarpa* seeds were obtained from closed capsules collected from one mature female *P. trichocarpa* tree in Moscow, ID in June 2019. The capsules were stored at 20 °C for two days. Seeds were aseptically removed from capsules and placed on *P. syringae pv. syringae* inoculum for five minutes at room temperature. Control seeds were placed on PDA and were not inoculated with *P. syringae pv. syringae*. In total, 50 seeds per treatment were transferred to moist, sterile filter paper in petri plates and incubated for 14 days at room temperature. Seed germination and seedling mortality were then recorded for each seed.

### 4.5. Data Analysis

All analyses were performed in R 3.6.3 [45], with figures generated using *tidyverse* 1.3 [46], *pals* 1.6 [47], *ggmap* 3.0 [48], and *cowplot* 1.1 [49]. We determined the total incidence of fungi, bacteria, and both fungi and bacteria for Oregon 2017, Idaho 2017, and Idaho 2018′s Clearwater River, ID and Moscow, ID locations. Isolation frequency of *Pseudomonas* was calculated as the number of fungi or bacteria isolated per tree out of the number of seeds collected per tree for the Idaho 2018 data. We used Pearson’s *X*^2^ to test for an association between seedling mortality and *Pseudomonas* emergence for the Idaho 2017 and Idaho 2018 data. We conducted Pearson’s X^2^ test of association for Idaho 2018 co-occurrence analyses, and a Fisher’s exact test if an expected value was less than 5 [50,51]. We used these tests to determine if there was an association between fungi and bacteria co-occurring in the same seed, and/or if there was an association between *Pseudomonas* and each fungal taxa isolated from the same seed. Due to varying sampling methods, quantitative comparisons of bacterial and fungal microbes across years and sites were not possible. Instead, we qualitatively compared the similarities and differences of microbial taxa and microbial incidence.

For the seedling mortality experiment, we measured seed germination and seedling mortality to determine if seed-borne *P. syringae pv. syringae* is a seedling pathogen of *P. trichocarpa*. For each treatment, seed germination rate was calculated by the number of seeds that germinated after 48 h by the total number of seeds. Seedling mortality was calculated by the number of seedlings that died within 24 h of germinating divided by the total number of seeds. Germination and seedling mortality were analyzed using Pearson’s *X*^2^ test of association.

## Figures and Tables

**Table 1:** Summary results for fungal and bacterial seed microbes of *Populus trichocarpa* for each sampling effort. Sampling location, number of trees, number of seeds collected per tree, if seed surface sterilization was used, the number of isolates and total incidence of fungi, bacteria, and both fungi and bacteria, and the percent microbes per seed are indicated.

					Total Incidence of Microbes			Percent of Seeds with Microbes		
Sampling Effort	Sampling Location	Number of Trees	Number of Seeds per Tree	Surface Sterilization	Fungi	Bacteria	Fungi and Bacteria	0	1	2+
Oregon 2017	Westport, OR	8	100	Yes	46 (5.8%)	0	0	94.2%	5.8%	0%
Idaho 2017	Moscow, ID	1	1050	No	56 (5.3%)	120 (11.4%)	5 (0.05%)	84%	15.3%	0.8%
Idaho 2018	Moscow, ID	1	60	No	15 (25%)	46 (76.7%)	9 (15%)	18.3%	63.3%	18.3%
Idaho 2018	Clearwater, ID	21	60	No	260 (20.6%)	353 (28%)	51 (4.1%)	56.7%	37.9%	5.3%

## Data Availability

Relevant files and script for data analyses are publicly available at www.github.com/sabrinaheitmann/ptri-seed-microbiota (accessed on 12 July 2017).

## Supplementary Materials

The following is available online at https://www.mdpi.com/article/10.3390/pathogens10060653/s1, Table S1: List of all sequenced fungal isolates from Oregon 2017 and Idaho 2018 sampling efforts, including information on isolate identification and collection.
