# Supplementary material for: Culturable Seed Microbiota of Populus trichocarpa
Source: Pathogens. 2021 May 24;10(6):653. doi: 10.3390/pathogens10060653 (PMC8225106; doi:10.3390/pathogens10060653)
Supplement: Supplementary file 1 [file pathogens-10-00653-s001.zip › pathogens-1208197-supplementary.pdf]

Table S1. List of all sequenced fungal isolates from Oregon 2017 and Idaho 2018 sampling efforts, including information on isolate identification and collection.

Isolate genus	Class	Collection site	Year	Source tree	GenBank accession
Aureobasidium	Dothideomycetes	Westport, OR	2017	B279	MT786275.1
Aureobasidium	Dothideomycetes	Westport, OR	2017	GW9857	MT786281.1
Aureobasidium	Dothideomycetes	Westport, OR	2017	GW9857	MT786289.1
Boeremia	Dothideomycetes	Westport, OR	2017	GW9765	MT786255.1
Boeremia	Dothideomycetes	Westport, OR	2017	GW9829	MT786263.1
Boeremia	Dothideomycetes	Westport, OR	2017	B328	MT786262.1
Botrytis	Leotiomycetes	Westport, OR	2017	B334	MT786279.1
Botrytis	Leotiomycetes	Westport, OR	2017	B334	MT786287.1
Cladosporium	Dothideomycetes	Westport, OR	2017	B261	MT786303.1
Cladosporium	Dothideomycetes	Westport, OR	2017	GW9765	MT786305.1
Cladosporium	Dothideomycetes	Westport, OR	2017	B868	MT786269.1
Cladosporium	Dothideomycetes	Westport, OR	2017	B868	MT786276.1
Cladosporium	Dothideomycetes	Westport, OR	2017	B328	MT786304.1
Colletotrichum	Sordariomycetes	Westport, OR	2017	B334	MT786278.1
Crocicreas	Leotiomycetes	Westport, OR	2017	B279	MT786285.1
Crocicreas	Leotiomycetes	Westport, OR	2017	B334	MT786254.1
Crocicreas	Leotiomycetes	Westport, OR	2017	B868	MT786273.1
Diaporthe	Sordariomycetes	Westport, OR	2017	B279	MT786286.1
Diaporthe	Sordariomycetes	Westport, OR	2017	B334	MT786295.1
Diaporthe	Sordariomycetes	Westport, OR	2017	GW9829	MT786296.1
Diaporthe	Sordariomycetes	Westport, OR	2017	B334	MT786282.1
Diaporthe	Sordariomycetes	Westport, OR	2017	B334	MT786297.1
Diaporthe	Sordariomycetes	Westport, OR	2017	B334	MT786257.1
Diaporthe	Sordariomycetes	Westport, OR	2017	B279	MT786290.1
Diaporthe	Sordariomycetes	Westport, OR	2017	B279	MT786280.1
Diaporthe	Sordariomycetes	Westport, OR	2017	B279	MT786292.1
Diaporthe	Sordariomycetes	Westport, OR	2017	B279	MT786301.1
Diaporthe	Sordariomycetes	Westport, OR	2017	B334	MT786283.1
Diaporthe	Sordariomycetes	Westport, OR	2017	B334	MT786291.1
Diaporthe	Sordariomycetes	Westport, OR	2017	B334	MT786258.1

Diaporthe	Sordariomycetes	Westport, OR	2017	B279	MT786259.1
Diaporthe	Sordariomycetes	Westport, OR	2017	B334	MT786267.1
Diaporthe	Sordariomycetes	Westport, OR	2017	B279	MT786284.1
Diaporthe	Sordariomycetes	Westport, OR	2017	B279	MT786288.1
Diaporthe	Sordariomycetes	Westport, OR	2017	B334	MT786298.1
Diaporthe	Sordariomycetes	Westport, OR	2017	GW9829	MT786299.1
Diaporthe	Sordariomycetes	Westport, OR	2017	B279	MT786302.1
Didymella	Sordariomycetes	Westport, OR	2017	B328	MT786265.1
Herpotrichia	Dothideomycetes	Westport, OR	2017	GW9857	MT786272.1
Alternaria	Dothideomycetes	Moscow, ID	2018	LOC67	MT786260.1
Alternaria	Dothideomycetes	Clearwater, ID	2018	SK6410	MT786264.1
Alternaria	Dothideomycetes	Clearwater, ID	2018	T257	MT786268.1
Alternaria	Dothideomycetes	Clearwater, ID	2018	SK966	MT786271.1
Alternaria	Dothideomycetes	Clearwater, ID	2018	SK962	MT786277.1
Asteroma	Sordariomycetes	Clearwater, ID	2018	T252	MT786266.1
Aureobasidium	Dothideomycetes	Clearwater, ID	2018	SK412	MT786274.1
Cladosporium	Dothideomycetes	Clearwater, ID	2018	T31510	MT786300.1
Cladosporium	Dothideomycetes	Clearwater, ID	2018	T3159	MT786294.1
Cladosporium	Dothideomycetes	Clearwater, ID	2018	SK6510	MT786256.1
Epicoccum	Dothideomycetes	Clearwater, ID	2018	SK965	MT786261.1
Epicoccum	Dothideomycetes	Clearwater, ID	2018	T1023	MT786270.1
Valsa	Sordariomycetes	Clearwater, ID	2018	SK163	MT786293.1
